# Correlation of electronic gadget usage, sleep quality & daytime sleepiness with working memory in Indian young adults

**DOI:** 10.3389/frsle.2026.1707437

**Published:** 2026-09-08

**Authors:** Ishan Gupta, Nasreen Akhtar

**Affiliations:** 1 All India Institute of Medical Science, New Delhi, India; 2 Department of Physiology, All India Institute of Medical Science, New Delhi, India

**Keywords:** day time sleepiness, screen time, sleep quality, working memory, young adults

## Abstract

**Aim:**

To assess the correlation of screen time, daytime sleepiness and quality of sleep with working memory in young adults.

**Methods:**

A cross sectional study of 119 young adults of the age group 18-30 years, who were administered a structured online questionnaire which collected details pertaining to basic socio denographic data, record of sleep wake activity and study habits, electronic gadget usage, working memory, as well as the standardized questionnaires Pittsburgh Sleep Quality Index (PSQI) and Epworth Sleepiness Scale (LSS).

**Results:**

Poor sleep quality which is indicated by a global PSQI score of more than was noted in 72/114 (6.3%) young adults Correlation of ISS score with working nomory was statistically significant with spearman's cho of 8.2607 The fitted regression model on multiple linear regression was: Working memory 68.8 0.779 (daily average screen time) + 8.55% (global PSQT score) 0.47 (ESS score) with the overall model being statistically significant [R28.073, Γ (3, 110)2.88, *p* < 0.05).

**Conclusions:**

Increased daytime sleepiness is correlated with a worse working memory among.

## 1 Introduction

Sleep is important to maintain homeostasis at all levels in the human body. With the advent of modern lifestyle, with excessive exposure to light till late in the night and erratic sleep patterns, there is an epidemic of adverse outcomes. Inadequate and interrupted sleep can lead to diabetes, cardiovascular diseases, accidents, depression, impaired cognition, risk of exhaustion/burnout, use of psychoactive substances and a poor quality of life (Lund et al., 2010; Fernández-Mendoza et al., 2009; Millman and Working Group on Sleepiness in Adolescents/Young Adults, 2005). This can also lead to poor academic performance among students (Machado-Duque et al., 2015). Academic performance correlates positively with working memory capacity in young adults, supported by multiple studies using GPA and standardized tests as markers (Alloway and Alloway, 2010). With the increasing use of different kinds of electronic gadgets, like smartphones, tablets, television, laptops, computers etc. and increasing use of internet for personal and professional use, the sleep patterns amongst youth have changed drastically in the recent years. Compulsive and excessive use of electronic gadgets during night hours among students affects their academic performance and family interaction. Although there have been previous studies highlighting the presence of insomnia, daytime sleepiness, shorter sleep duration and poor sleep quality in people working on night shifts, (Waage et al., 2014; Lin et al., 2012; De Martino et al., 2013) how screen time, quality of sleep, and daytime sleepiness interact to influence working memory, is unknown Further, ethno-geographic influences affecting these factors in young adults in India are unknown. To address this gap, the present study utilizes standardized questionnaires—Pittsburgh Sleep Quality Index (PSQI) (Buysse et al., 1989) to measure the quality of sleep young adults and Epworth Sleepiness Scale (ESS) to assess daytime sleepiness and examine how these variables, together with daily screen time, relate to working memory. The aim of the study was to assess the correlation of the average daily screen time usage, day-time sleepiness and quality of sleep quality with working memory in young adults.

## 2 Materials and methods

This was a cross-sectional study conducted among young adults belonging to the age group of 18–30 years. A total of 119 participants were recruited using convenience sampling. Sample size estimation was based on an expected correlation coefficient of 0.3 between sleep-related variables and academic performance, with 90% power and a 5% significance level, yielding a minimum sample size of approximately 108. We included 119 students, of whom, 114 met the eligibility criteria and completed the survey. The study was approved by the Institute Ethics Committee (IECPG-134/23.04.2020, RT-09/24.06.2020).

*Eligibility Criteria:*

*Inclusion Criteria:* Adults belonging to the age group of 18–30 years who are studying at an undergraduate or graduate level.

*Exclusion Criteria:* Students, who had any history of smoking and/or drug abuse, known sleep or psychiatric disorders or any other chronic diseases were excluded from the study. 5 students were excluded based on the eligibility criteria.

*Study tools:* The students were, following proper informed consent, administered a structured questionnaire which collected details pertaining to the following domains.

*Socio-demographic data:* Information pertaining to basic socio-demographic data was collected which included age, sex, height, weight, and record of sleep-wake activity reporting usual sleeping and waking-up times.

*Electronic gadget usage:* Information pertaining to electronic gadget usage was collected as self-reported data regarding the number of electronic gadgets used and the average screen time usage in a day. Also, information about the proportion of screen time involving interactive usage, usage in dark surroundings and usage involving texting/waiting for a reply was obtained. *Working memory:* In this study, academic performance is utilized as a surrogate marker of working memory. Previous studies have shown moderate correlation between the two (Alloway and Alloway, 2010). Information pertaining to self-reported average study hours as well as self-reported academic performance was collected. Academic performance was assessed by anonymized self-declared marks obtained in the last two internal university examinations and computing an average percentage of the same.

*Sleep quality:* Pittsburgh Sleep Quality Index (PSQI), a standardized questionnaire to assess the quality of sleep, was also administered. PSQI helps to evaluate quality of sleep, assessed by seven components which include subjective sleep quality, habitual sleep efficiency, sleep latency, sleep duration, sleep medication usage, sleep disturbances, as well as daytime dysfunction over the past month. Answers are scored on a four-point Likert Scale each scored from zero to three, where three reflects the negative extreme. A “poor” sleep quality is indicated by a global PSQI score of “5” or greater (Pilcher and Huffcutt, 1996).

*Daytime sleepiness:* Daytime sleepiness was assessed using another standardized questionnaire—Epworth Sleepiness Scale (ESS). ESS consists of eight questions, which are used to assess daytime sleepiness (Aderinto et al., 2024). A score of 10 or below falls in the normal range of sleepiness in healthy adults, whereas a score of 11 to 14 depicts mild sleepiness, 15 to 17 depicts moderate sleepiness and 18 to 24 depicts severe sleepiness.

*Statistical Analysis:* Data was analyzed using Stata-MP-16.0 (StataCorp, TX, USA) software. Categorical variables were expressed as numbers and percentages. Continuous variables were checked for approximate normality using the Shapiro Wilk test and were recorded as appropriate as mean, standard deviation or median, interquartile range. Correlation between continuous variables was checked using spearman's correlation. A student's *t*-test and analysisof-variance (ANOVA) were used to compare academic performance and categorical variables. A *p*-value of less than 0.05 was considered statistically significant. Linear regression analysis was done to evaluate if screen time, quality of sleep and daytime sleepiness predicted working memory.

## 3 Results

There were a total of 114 young adults with the age range 18–30 years (22.3 ± 3.9 years) with 61 males (53.5%). Body mass index ranged from 17.6 to 33.1 (23.4 ± 3.6 kg/m^2^). 91/114 students (79.8%) were medical students. Time to go to bed varied from 8:30 PM to 4:00 AM with 29/114 students going to bed after 2:00 AM. Time to get up varied from 4:00 AM to 2:00 PM with 10/114 students getting up after 10 AM. Total number of study hours varied from 1 to 56 h per week (20.5 ± 18.7 h per week).

The number of electronic gadgets used by students varied from 1–5 with 42 students using three or more than three electronic gadgets. The average daily screen time varied from 1 to 14 h (5.8 ± 2.8 h). Screen time of nine or more than 9 h per day was noted in 14/114 (13.2%) students. More than 60% of the screen time involving interactive usage (such as a smartphone or a video game console) was noted in 23/61 males and 13/53 females. Minority of the students spent more than 60% of their screen time in a dark room or in dark surroundings (2 males and 2 females) and a few spent more than 60% of their screen time in texting or waiting for a reply (4 males and 2 females).

Sleep quality was assessed by PSQI and a global PSQI score was calculated. The global PSQI score varied from 0 to 18 (6.9 ± 3.4). A global PSQI score of more than 5 (poor sleep quality) was noted in 72/114 (63%) students with 36 (59%) males and 36 (68%) females.

ESS score varied from 0 to 19 (7.0 ± 4.4). ESS score of more than 14 (moderate to severe daytime sleepiness) was noted in 9/114 (7.9%) students.

Working memory was derived via academic performance assessed by anonymized self-declared marks obtained in the last two internal university examinations and computing an average percentage. It varied from 40% to 83.5% (65%, 57.5%−72%).

Spearman's correlation was used to correlate working memory with other continuous variables. Correlation of ESS score with academic performance was statistically significant with spearman's rho of −0.2607 and a *p*-value of < 0.05. Other spearman's rho values and corresponding *p*-values are depicted in Table 1.

**Table 1:** Spearman's correlation between working memory and continuous variables.

Variables	Working memory	
	Spearman's rho	*p*-value
Age	−0.1806	0.0546
BMI	0.0499	0.5978
Study hours per week	−0.0114	0.9040
Daily screen time	−0.1599	0.0892
PSQI	0.0135	0.8866
ESS	−0.2607	0.0051^*^

^*^p value of < 0.05.

Student's *t*-test was used to compare working memory with categorical variables having 2 categories namely global PSQI score, sex, and phone ringer being kept on or off. No statistically significant result was found for global PSQI score or sex. However, between the groups of students keeping the phone ringer on vis-à-vis the group of students keeping the phone ringer off, the difference of the mean academic performance between the two groups was statistically significant [*t* = 2.18, df = 112, *p* = 0.0314].

Analysis-of-variance (ANOVA) was used to compare working memory with categorical variables having more than 2 categories namely ESS score, number of gadgets and percentage of screen usage being interactive/dark room-based/spent texting or waiting for a reply. None of the variables had a statistically significant result.

Simple linear regression analysis was done to evaluate if screen time, quality of sleep and daytime sleepiness independently predicted working memory in young adults. A *p*-value < 0.05 was found out for average daily screen time but not for global PSQI score or ESS score. In simple linear regression analyses, average daily screen time showed a statistically significant linear association with academic performance, whereas global PSQI and ESS scores did not. These findings should be interpreted as unadjusted linear associations rather than independent effects.

Multiple linear regression analysis was done to evaluate the interplay of screen time, quality of sleep and daytime sleepiness in affecting academic performance in young adults. The fitted regression model was: Working memory = 68.8-−0.77^*^(daily average screen time) + 0.55^*^(global PSQI score) – 0.47^*^(ESS score). Multiple linear regression including average daily screen time, global PSQI score and ESS score demonstrated a statistically significant overall model (*R*^2^ = 0.073, adjusted R^2^ = 0.047, *F*_(3, 110)_ = 2.88, *p* = 0.039). Average daily screen time was independently associated with lower academic performance (β = −0.77, 95% CI −1.52 to −0.02, *p* = 0.044), whereas global PSQI score (β = 0.55, 95% CI −0.22 to 1.31, *p* = 0.158) and ESS score (β = −0.47, 95% CI −1.05 to 0.12, *p* = 0.116) were not statistically significant.”

## 4 Discussion

Adequate and good quality sleep plays an important role in concentration, proper learning and good memory (Aderinto et al., 2024). Working memory is a multifaceted construct which can be influenced by various factors including sleep quality and usage of electronic gadgets among young adults The present study was conducted to examine the interplay of usage of electronic gadgets, quality of sleep, and daytime sleepiness in impacting working memory in young adults in India.

Daytime sleepiness, often a consequence of poor sleep quality, has been linked to reduced cognitive function and working memory among young adults. Working memory has been shown to have a moderate correlation with academic performance in previous studies (Alloway and Alloway, 2010). A previous study investigated the impact of sleep deprivation on cognitive performance and found that sleep-deprived individuals exhibited impaired cognitive function, which could adversely affect academic achievement (Pilcher and Huffcutt, 1996). A meta-analysis by Gomes et al. (2021) corroborated these findings, highlighting a significant negative association between sleep disturbances and academic performance (Gomes et al., 2021). In our study as well, we found a statistically significant negative correlation between working memory and ESS scores—a standardized questionnaire to assess daytime sleepiness.

Electronic gadgets have also led to increased screen time among young adults, which may have detrimental effects on their academic performance. Excessive screen time is often associated with disrupted sleep patterns, decreased concentration, and poor academic outcomes. A longitudinal study by Gentile et al. (2017) found that higher levels of screen time during adolescence were predictive of lower academic achievement in young adulthood (Gentile et al., 2017). A cross sectional study revealed a negative correlation between screen time before bedtime and academic performance among adolescents (Cain and Gradisar, 2010). Media exposure may adversely affect sleep through behavioral displacement and arousal-related mechanisms. Media use can encroach upon time allocated for sleep or sleep-promoting behaviors, while evening exposure may heighten autonomic and cognitive arousal, impairing pre-sleep relaxation. These effects collectively manifest as later sleep onset and shorter total sleep time with excessive media use (Cain and Gradisar, 2010). The pervasive use of electronic gadgets, such as smartphones and laptops, has become ubiquitous among young adults, posing challenges to their academic performance. Excessive gadget usage, particularly before bedtime, can disrupt sleep patterns and exacerbate daytime sleepiness. The impact of pre-sleep electronic media use on sleep habits and academic performance among adolescents has been studied and it was found that greater electronic media use was associated with poorer sleep quality and lower academic achievement (Van den Bulck, 2007). These findings underscore the need for strategies to mitigate the adverse effects of electronic gadget usage on academic performance. In our study, we found a negative correlation between academic performance and daily average screen time, but it was not statistically significant.

Sleep quality plays a pivotal role in determining academic success among young adults. Pittsburgh Sleep Quality Index (PSQI) is a standardized tool used for assessing quality of sleep, encompassing various dimensions such as duration of sleep, sleep latency, sleep efficiency, and sleep disturbances & medication usage. A study by Lund et al. (2010) utilized the PSQI to investigate the relationship between quality of sleep and academic performance among college students and found that poor sleep quality was associated with lower GPA scores. Additionally, another systematic review concluded that interventions aimed at improving sleep quality could lead to enhancements in academic performance (Dewald et al., 2010). Effective study habits are essential for academic success and are closely intertwined with sleep patterns and screen time (Gallego-Gómez et al., 2021). Young adults who adopt efficient study habits tend to achieve better academic outcomes compared to those with poor study habits (Gomes et al., 2021).

A longitudinal study by Crede and Kuncel (2008) examined the predictive validity of study habits on academic performance and found a significant positive correlation between effective study habits and GPA scores. Conversely, students with irregular study patterns and excessive screen time may experience difficulties in concentration and retention, ultimately leading to subpar academic performance.

Epworth Sleepiness Scale (ESS) is a standardized questionnaire used to assess daytime sleepiness, capturing an individual's propensity to doze off in various situations. On the other hand, the PSQI provides a comprehensive evaluation of sleep quality across multiple domains. Previous studies have demonstrated the reliability of the ESS in assessing daytime sleepiness and psychometric properties of the PSQI in evaluating sleep quality (Buysse et al., 1989; Johns, 1991; Suardiaz-Muro et al., 2023). These standardized questionnaires serve as valuable tools for researchers and clinicians to measure variables related to sleep and their impact on academic outcomes. In the present study, young adultswe came up with a multiple linear regression model where increased average daily screen time as well as increased daytime sleepiness predicted a worse academic performance. In the multiple linear regression model, the coefficient for global PSQI score was positive, indicating an unexpected direction of association after adjustment for screen time and daytime sleepiness. This finding should not be interpreted as evidence that poorer sleep quality improves academic performance. The unexpected direction may reflect sample-specific characteristics, residual confounding, or the shared variance among the sleep-related variables included in the model. Given the modest explanatory power of the model (*R*^2^ = 0.073), this finding should be interpreted cautiously and requires confirmation in larger and more representative cohorts.

A recent study from India highlighted the intricate dynamics of the influence of web streaming and screen time on the young Indian population (Chandra et al., 2023). It also emphasized the significance of adopting a well-rounded approach to digital engagement in policy-making and practical applications. High levels of stress in this population also makes the risk of obstructive sleep apnoea higher (Aggarwal et al., 2021).

The present study has a few shortcomings. All data was self-reported and may be subject to recall as well as social desirability bias. While bidirectional influences between academic performance and working memory exist, direct evidence for using academic performance as a reliable standalone marker remains limited, often requiring cognitive assessments for precision (Alloway and Alloway, 2010). The overall number of participants in the present study was less and a larger study involving more participants may highlight the effects of increased screen time, excessive daytime sleepiness and poor sleep quality on overall working memory of young adults.

In conclusion, in our cohort of young adults, daytime sleepiness was intricately linked to working memory in young adults. These findings underscore the value of daytime alertness as a functional indicator of academic outcomes. Interventions should prioritize strategies to improve alertness during daytime including regular bedtimes and reduced nocturnal electronic device usage. Future studies should adopt longitudinal study designs and incorporate chronotype, screen use context as well as more objective indicators of working memory to better understand the dynamics between technology, sleep and learning outcomes in young adults.

## Data Availability

The raw data supporting the conclusions of this article will be made available by the authors, without undue reservation.
